# Mitochondrial DNA and MitomiR Variations in Pancreatic Cancer: Potential Diagnostic and Prognostic Biomarkers

**DOI:** 10.3390/ijms22189692

**Published:** 2021-09-07

**Authors:** Loredana Moro

**Affiliations:** 1 Institute of Biomembranes, Bioenergetics and Molecular Biotechnologies, National Research Council, 70126 Bari, Italy; l.moro@ibiom.cnr.it; 2 Department of Biochemistry and Molecular Pharmacology, New York University Grossman School of Medicine, New York, NY 10016, USA; 3 Perlmutter NYU Cancer Center, New York University Grossman School of Medicine, New York, NY 10016, USA

**Keywords:** mitochondrial genome, pancreatic cancer, mitochondrial microRNAs

## Abstract

Pancreatic cancer is an aggressive disease with poor prognosis. Only about 15–20% of patients diagnosed with pancreatic cancer can undergo surgical resection, while the remaining 80% are diagnosed with locally advanced or metastatic pancreatic ductal adenocarcinoma (PDAC). In these cases, chemotherapy and radiotherapy only confer marginal survival benefit. Recent progress has been made in understanding the pathobiology of pancreatic cancer, with a particular effort in discovering new diagnostic and prognostic biomarkers, novel therapeutic targets, and biomarkers that can predict response to chemo- and/or radiotherapy. Mitochondria have become a focus in pancreatic cancer research due to their roles as powerhouses of the cell, important subcellular biosynthetic factories, and crucial determinants of cell survival and response to chemotherapy. Changes in the mitochondrial genome (mtDNA) have been implicated in chemoresistance and metastatic progression in some cancer types. There is also growing evidence that changes in microRNAs that regulate the expression of mtDNA-encoded mitochondrial proteins (mitomiRs) or nuclear-encoded mitochondrial proteins (mitochondria-related miRs) could serve as diagnostic and prognostic cancer biomarkers. This review discusses the current knowledge on the clinical significance of changes of mtDNA, mitomiRs, and mitochondria-related miRs in pancreatic cancer and their potential role as predictors of cancer risk, as diagnostic and prognostic biomarkers, and as molecular targets for personalized cancer therapy.

## 1. Introduction

Pancreatic cancer is one of the most aggressive types of malignancies. In the United States, the 5-year survival rate for people diagnosed with localized pancreatic cancer is 39%. This percentage drops to 13% for patients diagnosed with regional cancer, i.e., cancer that has spread to nearby structures or lymph nodes, and to 3% for people diagnosed with cancer that has metastasized to distant tissues/organs [1]. Pancreatic cancer can originate from two different types of cells: exocrine or endocrine cells [2]. The exocrine compartment includes the acinar cells that synthesize and secrete digestive enzymes, the cuboidal ductal cells that delineate smaller ducts, and the columnar ductal cells that delineate large pancreatic ducts which guide the digestive enzymes to the duodenum [3]. The endocrine cells represent 1–2% of the pancreas and form the islets of Langerhans [4]. Pancreatic endocrine neoplasms are less common and have a better prognosis. Instead, most pancreatic cancers are ductal adenocarcinomas (PDACs) [2,5]. PDACs are frequently diagnosed at an advanced stage and have a poor prognosis because of several factors, including nonspecific early symptoms, the intrinsic biological aggressiveness of PDAC, and the lack of effective therapies and specific biological markers for early diagnosis and risk prediction [6].

Only 15–20% of patients diagnosed with PDAC are eligible for surgical resection, the only potentially curative treatment for PDAC [6,7]. The remaining 80% of patients are diagnosed with locally advanced or metastatic PDAC and are ineligible for surgery. In patients with borderline or primary locally advanced pancreatic cancer, neoadjuvant therapy, including radiotherapy, chemotherapy, or radiochemotherapy, has gained importance in the last years as a strategy to downsize the tumor and thus allow a secondary resection to improve overall survival [8,9]. Pancreatic cancer is characterized by high heterogeneity and the presence of a dense desmoplastic tumor stroma that reduces the elasticity of the tumor tissue leading to an increase in tumor interstitial fluid pressure and consequent decrease in the rate of perfusion of chemotherapeutic drugs [10]. These factors contribute to the high chemo- and radiotherapy resistance of pancreatic cancer cells.

A number of clinical studies have demonstrated the improvement of PDAC patients’ survival when chemotherapy is administered both in the neoadjuvant and adjuvant setting compared with no chemotherapy [11]. However, the determination of the individual molecular profiles for personalized therapy has not been implemented yet in the clinical routine.

Recent studies have demonstrated an extensive reprogramming of cellular metabolism in PDAC, which would support cancer progression and chemoresistance [12,13,14]. The two pathways generating energy, glycolysis and mitochondrial respiration through oxidative phosphorylation (OXPHOS), coexist in PDAC [14], and the prosurvival role of mitochondria in pancreatic cancer stem cells has been demonstrated [15].

Mitochondria are cytoplasmic organelles acting as central players in regulating metabolism, apoptosis, calcium homeostasis, and cellular fate. Mitochondria possess their own genome, the mitochondrial DNA (mtDNA), which resides in the mitochondrial matrix. Each cell contains hundreds of mitochondria and thousands of mtDNA copies [16]. MtDNA is a maternally inherited circular DNA encoding 22 transfer RNAs, 2 ribosomal RNAs, and 13 proteins of the mitochondrial respiratory complexes and OXPHOS. In addition, mtDNA contains a noncoding regulatory region, the displacement loop (D-loop), essential for mtDNA replication and transcription and susceptible to mutations/alterations at high frequency, particularly in the hypervariable regions (HVs) [17]. Being maternally inherited without recombination events, mtDNA sequence variants can accumulate over time. A population of individuals that share the same mtDNA variants define a mitochondrial haplogroup, which can have a specific geographic location and is traceable through the maternal lineage. For instance, haplogroups L, L1, and L2 were found in sub-Saharan Africans; haplogroups T, U, V, W, and X were found in Europeans; and haplogroups H, I, J, and K were found in North Americans of European descent [18,19].

Several types of mtDNA alterations have been described in cancer cells. Among them, point mutations and alterations of the mtDNA content are the most common in cancer cells and may support cancer progression and therapy resistance in certain cancers [20,21,22,23].

MicroRNAs (miRNAs or miRs) are small noncoding RNAs (approximately 22 nucleotides) that regulate gene expression via mRNA degradation or translation inhibition by specifically binding to the 3′ untranslated region (UTR) of their target mRNAs [24,25]. About 50% of miRNA genes are located in cancer-associated genomic regions or in fragile sites, thus suggesting their role in cancer pathogenesis [24]. Indeed, miRNAs play an important role in the initiation and progression of cancer and may act as oncogenic miRNAs (also termed oncomiRs) if their upregulation results in inhibition of the target tumor suppressor gene, or tumor suppressor miRNAs if their downregulation increases the activity of a corresponding oncogene [26]. Notably, miRNAs profiles can differentiate normal tissue from cancer and can predict prognosis or response to therapy in several cancer types [26]. For instance, unique miRNA expression profiles not only distinguish normal B cells from malignant B cells in chronic lymphocytic leukemia but also are associated with factors that predict the clinical progression of the disease [27]. Notably, aberrant miRNA expression profiles have been described in several types of human cancers, suggesting that miRNAs have great potential as new diagnostic and prognostic biomarkers in cancer (reviewed in [28]). In addition, unique miRNA signatures can predict poor response to cancer therapy [29].

Mitochondrial microRNAs (mitomiRs) are a specific set of miRNAs that tightly orchestrate mitochondrial activity [30] and have become an emerging field of study in cancer research for their direct or indirect actions on mitochondria and their potential to predict response to therapy [31]. For instance, mitomiR-2392 partially inhibits mtDNA transcription, thereby downregulating ND4, COX1, and CYTB mRNA and protein levels and causing a metabolic switch from OXPHOS to glycolysis in tongue squamous cell carcinoma (TSCC) cells [32]. Intriguingly, low miR-2392 and high ND4, COX1, and CYTB levels correlated with increased sensitivity to cisplatin chemotherapy in TSCC. In addition, low ND4, COX1, and CYTB mRNA levels correlated with reduced overall survival not only in TSCC but also in PDAC and other cancer types [32], suggesting that mitomiR levels may predict chemosensitivity and prognosis.

This review summarizes and discusses the current knowledge on mtDNA and mitomiR alterations in PDAC and their potential use as diagnostic or prognostic biomarkers and as therapeutic targets. Finally, a section of this review highlights the current knowledge on changes of mitochondria-related miRNAs, i.e., miRNAs that modulate the expression of nuclear-encoded mitochondrial proteins, in PDAC.

## 2. mtDNA Alterations in PDAC: A Prognostic Marker?

A number of mtDNA alterations have been detected in human cancers. Among them, point mutations and copy number variations are the two most common changes in cancer [20,21,22,23]. Accumulation of mutations in mtDNA is about 10-fold greater than that in the nuclear genome due to close vicinity to ROS-generating sites, an error-prone replication, and a less efficient DNA repair system [20,21,22,23]. MtDNA encodes proteins of the respiratory chain complexes; thus, any pathogenic mutation affecting these proteins may potentially cause mitochondrial dysfunction and metabolic reprogramming, supporting cancer cell plasticity [33]. Notably, He et al. [34] detected widespread heteroplasmic mtDNA variants in normal human cells and a significant increase in homoplasmic and heteroplasmic variants in colorectal cancer. Intriguingly, the variants found in colorectal cancer were also detected in patients’ plasma, with important implications for cancer biomarker development. However, in pancreatic cancer, there are still controversies about the prevalence and significance of mtDNA changes in cancer development and progression owing to the limited number of studies in this field.

### 2.1. Changes in mtDNA Content

The mtDNA content has been reported to change in cancer compared to normal cells in a tissue- and tumor-specific manner. For instance, it is significantly reduced in breast cancer, but it is increased in papillary thyroid carcinomas [35]. A study that used a multiplex real-time PCR method for synchronized quantification of nuclear DNA and mtDNA in 43 resectable PDACs and 31 adjacent normal pancreatic tissue samples demonstrated that the mtDNA copy number is significantly reduced in cancer compared to normal tissue [36]. Furthermore, there was an inverse correlation between mtDNA content and increased malignancy as follows: mtDNA content in adjacent normal pancreatic tissue > low-grade > intermediate grade > high-grade cancer. However, the decrease in mtDNA copy number was not a significant prognostic marker for overall survival in resectable PDACs. Further studies with larger cohorts are needed to unravel the potential clinical relevance of reduced mtDNA content in PDAC patients.

### 2.2. mtDNA Haplogroups, Single-Nucleotide Polymorphisms (SNPs), and PDAC Risk

Specific haplogroups and SNPs have been reported to confer increased risk for certain cancer types, though controversies exist about the reproducibility of these associations (reviewed in [37]). In the context of PDAC, Navaglia et al. [38] analyzed the frequency of mtDNA somatic mutations in the D-loop region of 99 cases of pancreatic cancer, 42 cases of chronic pancreatitis, 18 cases of tumors of the pancreatic biliary tract, and 87 healthy controls. To discriminate between tumor-associated and germline mutations, mtDNA from blood samples of the same patients was sequenced. Somatic mutations were detected in 3/99 pancreatic cancer patients. The T16519C SNP was associated with the presence of diabetes mellitus, a comorbidity frequently found in pancreatic cancer patients, and with a worse prognosis. This variant may alter the transcription of mtDNA-encoded genes, resulting in OXPHOS dysfunction. Overall, this study suggests that somatic mutations in the D-loop region are rare in pancreatic cancer and cannot be considered a causative event. Notably, the T16519C SNP has been associated with an increased breast cancer risk [39]. Two subsequent studies in larger cohorts of pancreatic cancer patients excluded the involvement of the T16519C SNP in pancreatic cancer risk or prognosis [40,41]. Wang et al. [40] analyzed 24 mitochondrial SNPs in 955 primary PDACs from Caucasian patients and 1102 healthy Caucasian controls. None of the SNPs was associated with pancreatic cancer. In addition, evaluation of 10 core SNPs peculiar to the haplogroups H, I, J, K, T, U, V, W, and X (1719, 4580, 7028, 8251, 9055, 10,398, 12,308, 13,368, 13,708, and 16391) resulted in no significant association with pancreatic cancer. A subsequent study in 990 pancreatic cancer patients which genotyped 24 SNPs, including T16519C, confirmed the lack of association between mitochondrial SNPs or haplogroups and patients’ survival [41]. In 2012, Lam et al. [42] reported the sequences of the entire mtDNA in 286 pancreatic cancer cases and 283 controls from a large population-based study of pancreatic cancer in the San Francisco Bay Area. They found that five common variants were associated with pancreatic cancer, specifically *ND2* mt5460g (complex I), *COIII* mt9698c (complex IV), mt1811g (16S), mt12307g (tRNA), and mt150t (HV2). Furthermore, 19 haplogroup N/L-specific variants showed a statistically significant association with pancreatic cancer. Of these, 13 occurred in regions coding for complex I, III, IV, and V proteins, 2 in 12S rRNA, and 4 in HV or noncoding regions. Among the 13 coding-region variants, 2 caused nonsynonymous substitutions: K6N in CYTB, which likely belongs to a conserved sequence element, and L555Q in ND5, a mutation predicted to be probably damaging. In contrast with previous studies, the results reported by Lam et al. indicate that “aggregated common and rare variants” together with singleton variants (i.e., variants peculiar to a single patient) contribute to pancreatic cancer risk. Of note, this study included only a small sample size of patients of African and Asian ancestry, and thus it may not reflect pancreatic cancer risk for these specific ancestries.

### 2.3. mtDNA Mutations Accumulate in PDAC Metastases

A recent study used patient-derived cell lines to correlate mtDNA genotype to the phenotype in pancreatic cancer [43]. The authors sequenced the mtDNA and ~1000 nuclear genes encoding mitochondrial proteins and metabolic enzymes, and they identified 24 somatic mutations in the mtDNA and 18 mutations in the nuclear DNA of 12 patient-derived pancreatic cancer cell lines. Analysis of metabolic function in the context of these somatic mutations showed metabolic changes consistent with mitochondrial dysfunction (i.e., decreased oxygen consumption and increased glycolysis). Notably, the majority of somatic mtDNA mutations were found in subunits of complex I (ND1, ND2, ND4, ND5, ND6) and in noncoding regulatory regions. In addition, a few mutations occurred in COX1 (complex IV) and in CYTB (complex III). The authors proposed a model whereby pancreatic cancer cells reduce OXPHOS by positively selecting a number of somatic mitochondrial mutations, thus driving reductive glutamine metabolism, which, in turn, would provide biosynthetic building blocks to support cell proliferation. Of note, a recent analysis in primary PDAC cells demonstrated that a significant fraction (approximately 30%; 7/21 cases) of PDACs can be defined as “high OXPHOS” [44] and are characterized by enriched activity of complex I. From a translational point of view, these findings imply that a thorough characterization of the metabolic features of each PDAC may allow stratification of patients according to their metabolic profile and identify patients most likely to respond to mitochondrial respiration or glycolysis targeting. In this context, Masoud et al. [44] tested the complex I inhibitor phenformin on “high OXPHOS” PDAC cells and showed that phenformin cooperates with gemcitabine to eradicate “high OXPHOS” PDAC cancer cells.

A large-scale analysis of the mtDNA in 268 early-stage resected PDACs and paired nontumor tissues identified 304 mitochondrial somatic mutations, with at least one mutation in 61% of the patients [45]. The greatest proportion of mutations occurred in the noncoding control region (60/304). Frequently mutated were ND5, 12S rRNA, and COX1. In addition, 29 mutations in tRNAs were detected. Intriguingly, by analyzing mtDNA mutations in the primary tumors and metastases from six patients, the authors found that metastases have a higher number of mutations, suggesting that metastatic progression of PDAC may involve the accumulation of mtDNA mutations. This finding is in agreement with previous studies in other cancer types showing that mtDNA mutations or depletion may promote metastatic spreading [15,16,37].

Taken together, these studies indicate that certain mtDNA haplogroup variants may predispose to pancreatic cancer and that, similarly to other cancer types, accumulation of mtDNA mutations may support metastatic progression and, thus, may represent an adverse prognostic factor. A summary of the main findings of these studies is presented in Table 1.

## 3. MitomiR and Mitochondria-Related miR Variations in PDAC: Diagnostic, Prognostic, and Therapeutic Biomarkers

MicroRNAs (miRs) represent major epigenetic regulators, and their implications as potential diagnostic, prognostic, and therapeutic biological markers in pancreatic cancer have emerged in the last decade [46,47,48]. MitomiRs are a subset of microRNAs that tightly control mitochondrial functions. In 2011, Bandiera et al. [49] found 13 microRNAs enriched in the mitochondrial fraction of HeLa cells and termed them “mitomiRNAs”. In 2013, Ro et al. [50] identified an abundant population of small noncoding RNAs encoded directly by the mtDNA that control mitochondrial gene expression. However, several mitomiRs detected within mitochondria are transcribed in the nucleus and then imported into the mitochondrial matrix to regulate the expression of mitochondrial genes (reviewed in [51,52]). Nuclear-encoded mitomiRs and mitochondria-related miRs modulating the expression of mitochondria-localized proteins are a new major focus in the cancer field and are discussed here in the context of PDAC.

### 3.1. MitomiRs in PDAC

In pancreatic cancer, changes in the tissue levels of certain mitomiRs have been associated with chemoresistance and worse prognosis. miR-181c, a miRNA encoded by the nuclear genome, can suppress the expression of the mtDNA-encoded protein COX1 [52], thus affecting mitochondrial respiration. In pancreatic cancer, miR-181-c is significantly upregulated, correlates with a worse prognosis, and represses the Hippo pathway by directly repressing LATS2, MOB1, MST1, and SAV1, promoting pancreatic cancer cell survival and chemoresistance [53]. Though the authors did not analyze the mitochondrial functionality, it is likely that the prosurvival effect of high miR-181c levels may be in part mediated by miR-181c action on COX1.

Like miR-181c, miR-1 is another miRNA that can target both mtDNA- and nuclear DNA-encoded genes. COX1 and ND1 are two mtDNA-encoded proteins targeted by miR-1 [52]. Overexpression of miR-1 promotes translation of mitochondrial proteins, thereby supporting muscle differentiation [54]. In cancer cells, miR-1 works as a tumor suppressor and can target directly or indirectly a plethora of proteins, including MMP-2, MMP-9, and the antiapoptotic protein BCL2, which is considered one of the main targets of the tumor-suppressive action of miR-1 (reviewed in [55]). Thus, miR-1 acts both as mitomiR by targeting COX1 and ND1 and as mitochondria-related miR by targeting BCL2. Notably, forced expression of miR-1 suppresses the tumorigenic potential of cancer stem cells [56]. A study performed in 43 PDAC tissues and paired serum samples showed a significant downregulation of miR-1 in cancer tissue and sera of PDAC patients [57]. In addition, low miR-1 levels were associated with poor survival [57]. These preliminary results suggest that miR-1 could represent a novel PDAC diagnostic and prognostic marker. However, additional studies with larger patient cohorts are needed to confirm these findings.

miR-21 is one of the top miRNAs differentially expressed in PDAC vs. normal pancreas or chronic pancreatitis [58,59,60]. It promotes CYTB translation within mitochondria [61] and may target several nuclear-encoded genes, including the tumor suppressor PTEN [62] and RECK, a glycoprotein that negatively regulates matrix metalloproteinases, thus suppressing cancer invasion and metastasis [63]. miR-21 is deregulated in a number of solid tumors, including breast, papillary thyroid, colon, prostate, lung, cervical, head and neck, esophageal, and gastric cancers, as well as in hematological malignancies [64]. Ectopic expression of a miR-21 inhibitor in PDAC cells inhibits cancer cell proliferation, invasion, and metastasis [65,66] and promotes apoptosis and sensitivity to gemcitabine [66,67]. In the clinical setting, miR-21 was found to be overexpressed in PDAC [58], and its high levels were correlated with shorter patient survival both in metastatic and adjuvant settings [68], suggesting that it may represent a promising prognostic and therapeutic target for PDAC management. In addition, a recent study found elevated levels of miR-21 in the exosomes from the peripheral blood of PDAC patients [69], suggesting that miR-21 may also be candidate as an early diagnostic biomarker for PDAC.

A summary of the mitomiRs analyzed in pancreatic cancer and their potential clinical impact is reported in Table 2.

### 3.2. Mitochondria-Related miRs in PDAC

Besides mitomiRs that directly control the mitochondrial functionality by modulating the expression of mtDNA-encoded proteins, a number of miRs can affect the mitochondrial activity by targeting nuclear-encoded mitochondrial proteins (reviewed in [84]), thereby modulating chemoresistance and cancer progression.

miR-31 has a plethora of targets and can function as tumor suppressor or as oncomiRNA depending on the cancer type (reviewed in [85]). In PDAC cell lines resistant to gemcitabine and p53-mutated, miR-31 is downregulated, and its suppression correlates with overexpression of the antiapoptotic, outer mitochondrial membrane protein BCL2 [71], indicating that miR-21 may modulate PDAC chemoresistance through BCL2.

Intriguingly, BCL2 is also the target of miR-345 [73] and miR-34a [77], two of the most significantly downregulated miRNAs in pancreatic cancer [58,70,74,75,76,86]. Forced expression of miR-345 in PDAC cell lines induced apoptosis, accompanied by upregulation of BCL2, loss of mitochondrial membrane potential, activation of caspase 3/7, and PARP-1 cleavage [76], suggesting that miR-345 restoration could be exploited for PDAC therapy. In addition, miR-345 downregulation was correlated with PDAC progression [73]; thus, it may represent a prognostic biomarker for pancreatic cancer.

miR-34a, another miRNA having BCL2 as a direct target [77], is silenced through epigenetic mechanisms in many cancers, including PDAC [87]. Notably, loss of miR-34a in PDAC patients correlates with poor prognosis, and miR-34a serum levels have been proposed as PDAC diagnostic biomarkers [70,74,75,76]. Preclinical studies support the therapeutic potential of miR-34a in combination therapy: in mice bearing PDAC tumors, amphiphilic nanocarrier delivery of miR-34a mimics, together with PLK1 siRNA, ameliorated the therapeutic response [78]. Furthermore, a mouse model of pancreatic conditional deletion of miR-34a (*Kras^G12D^*; *Mir34a*^Δ/Δ^) revealed the presence of preneoplastic lesions and PDAC development earlier than in *Kras^G12D^* control mice [79]. This protumorigenic effect was accompanied by an early increase in normal pancreatic acinar cells of TNF-α and interleukin-6, two proinflammatory cytokines, and by recruitment of immune cells in the microenvironment [79]. In addition, miR-34a restoration in pancreatic cancer cell lines reduced the number of cancer stem cells, tumorsphere formation in vitro, and tumor development in vivo [80], further confirming that miR-34a represents a PDAC tumor suppressor.

A study performed in human pancreatic cell lines demonstrated that miR-491-5p targets Bcl-XL, an antiapoptotic protein of the BCL2 family [88] localized to the outer mitochondrial membrane where it binds BAX, thereby inhibiting BAX-induced outer membrane permeabilization [88]. Notably, Bcl-XL is also present in the inner mitochondrial cristae where it stabilizes the mitochondrial membrane potential, thus supporting mitochondrial energy balance during stress conditions [89]. Guo et al. [81] have shown that overexpression of miR-491-5p in a pancreatic cancer cell line reduced Bcl-XL and TP53 protein levels. In addition, miR-491-5p expression activated the intrinsic mitochondrial apoptotic pathway independently of TP53, suggesting a preponderant role of Bcl-XL in miR491-5p-mediated apoptosis. To date, the in vivo relevance of this study is still not available. Future studies aimed at characterizing miR-491-5p expression in PDAC tissues and patients’ plasma/serum may shed light on the potential translational significance of miR-491-5p levels in PDAC management.

Two recent studies performed in pancreatic cancer cell lines suggest that miR-125a may represent a PDAC tumor suppressor miR [82,83]. Yao and colleagues [82] reported that miR-125a levels are elevated in chemoresistant SW1990GZ pancreatic cancer cells compared to parental SW1990 cells. In addition, while suppression of miR-125a in SW1990GZ cells increased sensitivity to gemcitabine, its overexpression increased chemosensitivity in SW1990 cells. In PANC-1 pancreatic cancer cells, miR-125a activated mitochondrial fission by targeting mitofusin 2 (Mfn2)—a protein localized in the outer mitochondrial membrane required for mitochondrial fusion—thereby triggering mitochondria-mediated apoptosis and impairing cancer cell invasion [83]. miR-125a is downregulated in several cancer types, including ovarian cancer [90], gastric cancer [90], and breast cancer [91]. However, as discussed above for miR-491-5p, the in vivo relevance and the potential value of miR-125a as prognostic and/or diagnostic biomarker in PDAC remain to be determined. Table 2 includes a summary of the main findings on the clinical significance of changes in mitochondria-related miRs in PDAC.

## 4. Conclusions

PDAC is one of the most deadly cancer types, owing to difficulties in early diagnosis and its intrinsic aggressive biology and chemoresistance. Cancer research on mitochondria has gained momentum in recent years because of the pivotal role of mitochondria as mediators of apoptosis and chemoresistance. Several mitochondrial changes occur during cancer development and progression: some of these modifications act directly at the level of mtDNA, while others involve nucleus-encoded mitochondrial proteins and RNA. In PDAC, specific mtDNA mutations or SNPs may increase cancer risk, and a high number of mtDNA mutations may support an invasive phenotype. Notably, modifications in mitomiR and mitochondria-related miR levels have been implicated in PDAC chemoresistance through epigenetic changes in the expression levels of key mitochondrial antiapoptotic proteins. Recent studies reported in this review indicate that certain mitomiRs and mitochondria-related miRs could be further exploited as PDAC diagnostic or prognostic biomarkers and as therapeutic targets, which could pave the way for development of effective personalized treatments. Undoubtedly, more preclinical and clinical studies are needed to better define the role of mitomiRs and mitochondria-related miRs in cancer and expedite the translation into clinical practice.

## Figures and Tables

**Table 1:** mtDNA changes in pancreatic cancer and clinical significance.

mtDNA Region Analyzed	Analysis	Samples	Findings and Clinical Correlations	References
Whole mtDNA	mtDNA content	43 resectable PDACs and 31 adjacent normal pancreatic tissues	mtDNA depletion detected in pancreatic cancer. mtDNA content inversely correlated with tumor grade. No correlation with patient survival.	[36]
D-loop	Mutations	99 cases of pancreatic cancer, 42 cases of chronic pancreatitis, 18 cases of tumors of the pancreatobiliary tract, and 87 healthy controls	3/99 pancreatic cancer patients displayed mutations. T16519C SNP correlated with diabetes mellitus and worse prognosis.	[38]
D-loop 12S rRNA 16S rRNA ND2 ND3 ND4 ND5 COI COII CYTB ATPase6 tRNA	24 SNPs, including T16519C	955 primary pancreatic adenocarcinomas from Caucasian patients and 1102 healthy Caucasian controls; 990 pancreatic cancer patients	No association of the 24 SNPs with pancreatic cancer risk or survival.	[40,41]
Whole mtDNA	Mutations	286 pancreatic cancer cases and 283 controls	ND2 mt5460g (complex I), COIII mt9698c (complex IV), mt1811g (16S), mt12307g (tRNA), and mt150t (HV2) associated with pancreatic cancer; 19 haplogroup N/L-specific variants showed a statistically significant association with pancreatic cancer.	[42]
Whole mtDNA + ~1000 nuclear genes encoding mitochondrial proteins and metabolic enzymes	Mutations	12 patient-derived pancreatic cancer cell lines	24 mtDNA somatic mutations and 18 nuclear DNA mutations were identified. Mutations were phenotypically associated with mitochondrial dysfunction.	[43]
Whole-genome sequencing	Mutations	268 early-stage resected PDACs and paired nontumor tissues;for 6 patients, primary tumor and metastasesanalyzed	304 mtDNA somatic mutations, with at least 1 mutation in 61% of the patients.60/304 mutations in the noncoding region. Metastases have a higher number of mtDNA mutations and thus may represent an adverse prognostic marker.	[45]

**Table 2:** MitomiR and mitochondria-related miR changes in pancreatic cancer and their clinical significance.

MitomiR/ Mitochondria-Related miR *	Targeted Mitochondrial Protein	Samples	Findings and Clinical Potential	References
miR-181c	COX1	124 pancreatic cancer samples and 10 noncancerous pancreatic tissues. PANC-1 and BxPC3 pancreatic cancer cell lines.	High miR-181c levels in pancreatic cancer samples. Elevated miR-181c predicted poor patient overall survival. Potential therapeutic target and prognostic marker.	[52,53]
miR-1 **	COX1, ND1, BCL2	43 PDAC tissues and paired serum samples	Low miR-1 levels associated with poor survival of PDAC patients. May represent a novel PDAC diagnostic and prognostic marker.	[52,57]
miR-21	CYTB	65 PDACs and matched benign adjacent pancreatic tissue + 42 chronic pancreatitis tissues. 81 PDAC patients and normal ductal samples + 7 PDAC cell lines, 7 primary cultures, fibroblasts, and a normal pancreatic ductal cell line. Peripheral blood plasma of 36 patients with pancreatic cancer and 65 healthy controls. Serum and salivary samples from 24 patients with PDAC and 10 healthy controls. 14 cancer cell lines, primary cultures of normal pancreatic epithelial cells and fibroblasts, a human normal pancreatic ductal epithelial cell line + 25 pancreatic cancer tissue samples and 25 pancreatic normal tissues. 7 pancreatic cancer lines and 1 normal pancreatic ductal precursor cell line.	High levels of miR-21 in PDAC. High levels in the exosomes isolated from peripheral blood of PDAC patients.Elevated miR-21 levels correlate with shorter patients’ survival, both in the metastatic and adjuvant setting. Ectopic expression of a miR-21 inhibitor in PDAC cells inhibits cancer cell proliferation, invasion, and metastasis and promotes apoptosis and sensitivity to gemcitabine.May represent a therapeutic target, a diagnostic biomarker, and an adverse prognostic marker.	[58,59,60,61,65,66,67,68,69,70]
miR-31	BCL2	PDAC cell lines MIA-PaCa-2, PANC-1, BxPC-3, SU.86.86, and AsPC-1 and gemcitabine-resistant PANC-1 (PANC-1-GR) and MIA-PaCa-2 (MIA-PaCa-2-GR).	PDAC cell lines resistant to gemcitabine and p53-mutated show decreased miR-31 levels. miR-31 suppression may promote PDAC chemoresistance by upregulating BCL2, an antiapoptotic protein.	[71]
miR-345	BCL2	28 pancreatic cancers, 6 normal pancreas and 15 adjacent benign tissues + the pancreatic cancer cell lines Panc-1, HS766T, MIA PaCa-2, HPAF-II, BxPC-3, Mpanc-96, PL45, Panc03.27, and Panc10.05. Human pancreatic cancer cell lines (MiaPaCa, Panc1, Colo-357, HPAF, ASPC-1, Panc10.05, Panc02.03, Panc03.27, BXPC3, CFPAC, CAPAN1, and SW1990) + normal immortalized pancreatic cell line hTERT-HPNE and its progressively malignant derivatives + normal (n = 7) and cancerous (n = 21) pancreatic tissues.	miR-345 is downregulated in PDAC (fold change: −14.5). miR-345 loss correlates with PDAC progression. Forced expression of miR-345 induces apoptosis in PDAC cell lines. miR-345 loss may represent a negative prognostic biomarker.miR-345 restoration could be exploited in PDAC therapy.	[72,73]
miR-34a	BCL2	159 patients with PDAC tumors (serum + tissue), 82 patients with benign pancreatic lesions (serum + tissue), and 44 age- and gender-matched healthy subjects (serum). Serum and salivary samples from 24 patients with PDAC and 10 healthy controls. PDAC tissue from 48 patients.PDAC tissues from 90 patients with or without gemcitabine treatment after resection of pancreatic cancer + two gemcitabine-resistant pancreatic cancer cell lines. 10 PDAC specimens + MiaPaCa2, BxPC3, and Panc1 pancreatic cancer cells lines + PDAC MiaPaCa2 xenografts. PDAC mouse model of pancreatic-specific deletion of miR-34a (KrasG12D; Mir34aΔ/Δ) and control mice (KrasG12D). p53-mutant human pancreatic cancer cell lines MiaPaCa2 and BxPC3.	Loss of miR-34a in PDAC patients correlates with reduced survival. In gemcitabine-treated PDAC patients, overall survival time was significantly dependent on both miR-34a expression and lymph nodes status; in the non-gemcitabine group, miR-34a expression was an independent prognostic marker for pancreatic cancer patients with a relative risk of 2.920. In a PDAC mouse model of pancreatic-specific deletion of miR-34a (KrasG12D; Mir34aΔ/Δ) preneoplastic lesions and PDAC developed earlier than in KrasG12D control mice. miR-34a restoration in PDAC cell lines reduces the number of cancer stem cells, tumorsphere formation in vitro, and tumor development in vivo. miR-34a serum levels have been proposed as PDAC diagnostic biomarkers.	[70,74,75,76,77,78,79,80]
miR-491-5p	Bcl-XL	Human PDAC cell lines SW1990, MiaPaCa-2, Capan-1, and AsPC-1 + 1 normal pancreas	Overexpression of miR-491-5p in pancreatic cancer cells reduced Bcl-XL and TP53 protein levels and activated the intrinsic mitochondrial apoptotic pathway.	[81]
miR-125a	Mfn2	Parental and gemcitabine-resistant SW1990GZ pancreatic cancer cells. PANC-1 pancreatic cancer cell line.	miR-125a levels are elevated in chemoresistant SW1990GZ cells. In PANC-1 cells, miR-125a activated mitochondrial fission by targeting Mfn2, thereby triggering mitochondria-mediated apoptosis and impairing cancer cell invasion.	[82,83]

* “Mitochondria-related miR” refers to a miR that modulates the expression of a nuclear-encoded mitochondrial protein. ** miR-1 acts both as mitomiR by targeting COX1 and ND1 and as mitochondria-related miR by targeting BCL2.
